# Similar Levels of X-linked and Autosomal Nucleotide Variation in African and non-African populations of *Drosophila melanogaster*

**DOI:** 10.1186/1471-2148-7-202

**Published:** 2007-10-25

**Authors:** Nadia D Singh, J Michael Macpherson, Jeffrey D Jensen, Dmitri A Petrov

**Affiliations:** 1 Department of Biological Sciences, Stanford University, Stanford, CA 94305 USA; 2 Department of Molecular Biology and Genetics, Cornell University, Ithaca NY 14853 USA

## Abstract

**Background:**

Levels of molecular diversity in Drosophila have repeatedly been shown to be higher in ancestral, African populations than in derived, non-African populations. This pattern holds for both coding and noncoding regions for a variety of molecular markers including single nucleotide polymorphisms and microsatellites. Comparisons of X-linked and autosomal diversity have yielded results largely dependent on population of origin.

**Results:**

In an attempt to further elucidate patterns of sequence diversity in *Drosophila melanogaster*, we studied nucleotide variation at putatively nonfunctional X-linked and autosomal loci in sub-Saharan African and North American strains of *D. melanogaster*. We combine our experimental results with data from previous studies of molecular polymorphism in this species. We confirm that levels of diversity are consistently higher in African versus North American strains. The relative reduction of diversity for X-linked and autosomal loci in the derived, North American strains depends heavily on the studied loci. While the compiled dataset, comprised primarily of regions within or in close proximity to genes, shows a much more severe reduction of diversity on the X chromosome compared to autosomes in derived strains, the dataset consisting of intergenic loci located far from genes shows very similar reductions of diversities for X-linked and autosomal loci in derived strains. In addition, levels of diversity at X-linked and autosomal loci in the presumably ancestral African population are more similar than expected under an assumption of neutrality and equal numbers of breeding males and females.

**Conclusion:**

We show that simple demographic scenarios under assumptions of neutral theory cannot explain all of the observed patterns of molecular diversity. We suggest that the simplest model is a population bottleneck that retains an ancestral female-biased sex ratio, coupled with higher rates of positive selection at X-linked loci in close proximity to genes specifically in derived, non-African populations.

## Background

*Drosophila melanogaster *has been extensively used to investigate the role of various evolutionary forces in the generation and maintenance of sequence variation in natural populations. *D. melanogaster *is a cosmopolitan species believed to be African in origin [1] and has only recently colonized the rest of the world. This demographic history makes *D. melanogaster *an attractive system for assessing the impact of factors such as demography and natural selection on levels of extant sequence variation.

Studies of molecular polymorphism in *D. melanogaster *abound (for review see [2]). However, despite plentiful polymorphism data, the history of selection and demography of *D. melanogaster *remains relatively obscure. Some of the difficulty stems from the fact that the current data are biased in favor of genic (synonymous sites, UTRs, and intronic) and mostly X-linked loci (for review see [2]). While there have been some studies of intergenic sequence polymorphism, most of the studied regions are in close proximity to genes [3-5]. In addition, features such as local mutation rate and local recombination rate, both of which are likely to influence patterns of polymorphism, have not been controlled for in previous studies. As a result, patterns of polymorphism at these loci are likely to reflect not only genome-wide processes such as demographic history but also local, locus-specific effects of selection, mutation rate variability and Hill-Robertson [6,7] effects.

All studies to date do support a general reduction in levels of variation in derived versus ancestral strains of *D. melanogaster*. This was initially reported in studies relying on electrophoretic variants of proteins as well as variations in restriction maps (*e.g*. [8-11]); more recently single nucleotide polymorphism and microsatellite data have been used to show similar patterns (for review see [2]). Within these studies of nucleotide or microsatellite variability, however, the magnitude of this reduction appears to be heavily dependent on the choice of loci. Initial reports investigating X-linked loci consistently showed reductions in sequence variation in non-African, derived strains [9,12,13], while autosomal loci appeared to have more variable reductions in sequence polymorphism in non-African strains relative to their African progenitors [14-21]. More recent, large-scale studies show similar trends; sequence variation is markedly reduced on the X chromosome in derived strains while reductions in diversity at autosomal regions are less uniform across loci [22,23]. These results were generally interpreted as evidence that purely demographic processes could not fully account for patterns of nucleotide variability in non-African populations of *D. melanogaster*. Multilocus studies evaluating the frequency distributions of nucleotide variants also seem to implicate selective forces in the generation of patterns of nucleotide variability in these derived populations [3,4,24], as do studies using microsatellites [25-29].

Because patterns of variation show locus-specific effects, direct comparisons of diversity levels between the X chromosome and the autosomes have also shown variable results. Some studies suggest that X-linked diversity is indeed lower than autosomal diversity in *D. melanogaster *and its cosmopolitan sister species *D. simulans *[27,29]. However, the suggestion has also been made that while this is the case for non-African strains of these species, X-linked diversity is equal to or even greater than autosomal diversity in African strains [22,28]. There are also some data to support increased levels of variation of autosomal loci in African and some non-African strains but not in others [23].

It is thus clear that sampling plays an important role in the studies of molecular polymorphism in Drosophila. To shed more light on X-linked and autosomal diversity in ancestral and derived strains of *D. melanogaster*, we conducted a careful survey of single nucleotide polymorphism in *D. melanogaster*. We chose loci so as to minimize the potential effects of locus-specific forces that affect nucleotide variation such as selective constraint on protein function, Hill-Robertson effects, and variation in mutation rates. The chosen loci are intergenic, located in regions of high recombination, have a similar GC content, are as far as possible from genes, and have a similar level of divergence with *D. simulans*. By using stringent and uniform criteria for candidate locus selection, we hoped to minimize locus-specific effects such that we might be able to compare autosomal and X-linked diversity between ancestral and derived strains in a consistent manner.

Our findings indicate that levels of nucleotide diversity in North American strains of *D. melanogaster *are reduced compared to those in African strains, with similar reductions in diversity at the X-linked and autosomal loci. Moreover, levels of X-linked and autosomal diversity are more similar than expected under an assumption of strict neutrality and an equal sex ratio in both ancestral and derived populations of *D. melanogaster*. If we assume that levels of sequence diversity in extant African populations are indicative of levels of diversity in ancestral populations, these data are consistent with an historical excess of breeding females over the number of breeding males and a population bottleneck where this excess is retained. These findings are particularly relevant to the observation of consistently higher codon bias of X-linked genes in *D. melanogaster *[30-32] and may imply that natural selection might be more efficient at X-linked loci in general.

We also combined our data with much available published single nucleotide polymorphism data and conducted a meta-analysis. Results from this analysis suggest that while levels of diversity in African strains are greater than diversity levels in non-African strains, this effect is much more pronounced on the X chromosome than it is on the autosomes. In addition, the compiled data from ancestral populations suggest that levels of nucleotide polymorphism are similar between X-linked and autosomal loci. If we again use extant sequence diversity in African populations as indicators of ancestral polymorphism, these data cannot be fully explained by a population bottleneck that retains the presumed unequal ancestral sex ratio.

We discuss the differences between the two datasets and consider several hypotheses aimed at reconciling the observed patterns. While the data from the intergenic loci presented here are consistent with a female-biased sex ratio in ancestral and derived populations coupled with a population bottleneck, the results from the meta-analysis are qualitatively consistent with either a model wherein the ancestral population has a female-biased sex ratio that is not retained during and after the bottleneck, or where the ratio of the effective sizes of the X and autosomes is less than one in the ancestral population and this ratio is preserved during and after a bottleneck. Presently, it appears that a demographic model incorporating a female-biased sex ratio in the ancestral population and a population bottleneck is likely to be the most appropriate. However, our confidence in this model would benefit from further consistent and careful sampling of polymorphism in the *D. melanogaster *genome, particularly on the autosomes.

## Results

### Candidate locus selection

We identified eight autosomal and eight X-linked loci in the *D. melanogaster *genome based on several criteria designed to minimize the effects of natural selection on levels of sequence variation. We chose regions of the genome that are nongenic and located as far from genes as possible. The chosen loci are at least 5 kb and on average 21 kb from the nearest annotated gene as measured from the midpoint of the candidate locus to the 5' or 3' end (depending on the orientation) of the nearest coding sequence. They ranged in size from 322 to 487 bp (Table 1). All the studied loci are located in high recombination regions of the genome [33] to minimize linkage to functionally important sites. We computed divergence with *D. simulans *and picked regions with similar rates of substitution to control for both selective constraint and mutation rate variation as much as possible. Using these criteria, we selected loci least likely to be affected by natural selection acting either on the loci themselves or on nearby linked loci, and we controlled for variation in recombination rate and mutation rate as much as possible.

**Table 1:** Summary statistics of X-linked and autosomal loci

Genomic Location	Number of Strains (NA^a^/NF^b^)	Number of Sites (NA^a^/NF^b^)	Divergence^c^	θ_s_^d ^NA^a^	θ_s_/Divergence^e ^NA^a^	Tajima's D NA^a^	θ_s_^c ^AF^b^	θ_s_/Divergence^e ^AF^b^	Tajima's D AF^b^
2R	12/12	486/411	0.06	9.05 (4.49–14.12)	1.38 (0.69–2.39)	0.51	5.04 (1.93–9.01)	0.78 (0.24–1.53)	1.07
2R	11/12	449/482	0.05	8.45 (4.10–13.64)	2.92 (1.11–5.94)	0.43	8.50 (3.93–13.11)	3.00 (1.24–6.08)	-0.79
2L	11/11	325/487	0.06	5.65 (2.10 – 9.82)	1.01 (0.35–1.98)	-0.79	13.21 (7.70–19.59)	2.40 (1.23–4.03)	-0.77
2L	11/12	458/424	0.08	8.81 (4.19 – 13.78)	1.91 (0.85–3.52)	-0.13	14.45 (9.17–20.90)	2.99 (1.61–5.26)	-0.93
3R	12/9	402/474	0.06	7.65 (3.50 – 12.79)	1.27 (0.56–2.36)	-0.75	24.30 (15.93–32.62)	3.96 (2.33–6. 40)	-0.84
3R	12/11	322/329	0.06	14.49 (8.65 – 20.96)	2.78 (1.38–4.89)	-0.53	15.64 (9.57–22.07)	2.98 (1.60–5.32)	0.31
3L	11/12	444/462	0.07	10.24 (5.14 – 15.42)	2.16 (1.07–3.89)	-0.48	36.27 (27.55–46.00)	7.56 (4.66–11.95)	1.02
3L	12/12	412/365	0.06	9.46 (4.39 – 15.35)	1.97 (0.92–3.48)	-0.12	25.88 (17.77–34.15)	5.36 (3.07–9.15)	-1.46
X	12/11	356/394	0.07	16.24 (10.61–22.72)	4.35 (2.13–8.12)	0.67	18.43 (11.63–25.82)	4.93 (2.49–9.18)	-1.12
X	12/11	391/385	0.08	5.49 (1.57 – 9.44)	0.87 (0.31 – 1.70)	0.33	16. 41 (9.85–23.28)	2.61 (1.33–4.65)	0.88
X	10/10	346/485	0.05	5.01 (0.91 – 10.14)	1.45 (0.32–3.00)	0.015	12.53 (7.64–18.79)	3.68 (1.85–7.12)	-1.02
X	12/11	447/406	0.09	15.69 (9.29–22.79)	3.20 (1.60–5.59)	0.74	39.06 (28.89–48.99)	7.95 (4.81–12.95)	-0.30
X	12/11	383/355	0.12	5.97 (1.72–11.18)	0.48 (0.16–0.94)	-0.49	28.28 (18.56–37.94)	2.22 (1.35–3.47)	-1.21
X	12/10	458/481	0.07	15.05 (9.13–22.23)	2.32 (1.21–3.93)	1.25	15.94 (9.69–22.31)	2.52 (1.40–4.12)	-.0.42
X	12/12	474/471	0.03	0.68 (0 – 2.09)	0.56 (0.23–1.63)	-1.14	9.01 (4.84–14.52)	5.28 (2.00–12.95)	-1.66
X	12/12	441/410	0.03	2.19 (0–5.17)	1.08 (0.23–2.77)	–0.38	2.24 (0–5.15)	1.09 (0.23–3.21)	-0.83
Average Overall				9.00 (7.70–10.20)	1.72 (1.46–2.00)		17.52 (15.73–19.17)	3.36 (2.96–3.76)	
Average autosomal				9.39 (7.75–11.08)	1.79 (1.39–2.19)		17.79 (15.24–20.24)	3.35 (2.78–4.03)	
Average X linked				8.51 (6.90–10.29)	1.67 (1.27–2.13)		17.30 (15.02–19.93)	3.38 (2.74–4.13)	

^a ^North American

^b ^African

^c ^Divergence with *D. simulans*

^d^θ_*s *_× 10^3 ^with (95% confidence interval)

^e^θ_*s *_/Divergence × 10 with (95% confidence interval

### Polymorphism and divergence at intergenic loci

**Figure 1:**
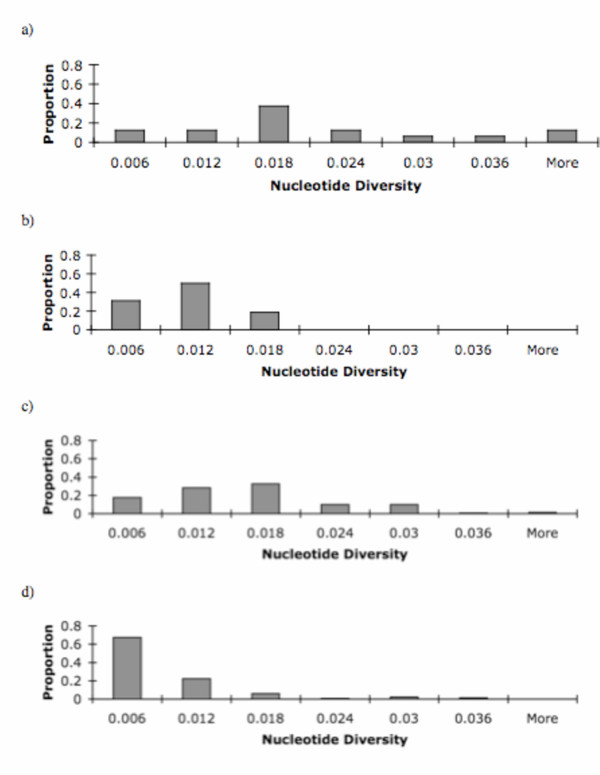
Frequency distribution of observed nucleotide diversity across loci in a) Singh *et al*. 16 X-linked and autosomal loci from African strains, b) Singh *et al*. 16 X-linked and autosomal loci from North American strains c) compiled data from X-linked and autosomal loci for African strains and d) compiled data from X-linked and autosomal loci for African strains.

We surveyed these sixteen noncoding loci for levels of nucleotide diversity using twelve African strains and twelve North American strains of *D. melanogaster *(see Methods). Sequence data were collected from between nine and twelve strains per locus for each population. Levels of nucleotide diversity estimated based on the number segregating sites (θ_s_) are presented in Table 1 and Figure 1. To test for selective neutrality at each locus, Tajima's *D *[34] was computed, and in all cases was not significantly different from zero (*P *> 0.05, all loci). Tajima's *D *for these data grouped by population or chromosome was also not significantly different from zero (*P *> 0.2, sign test, both cases).

Average levels of nucleotide diversity per site (estimated by θ_s_) were 17.52 × 10^-3 ^(15.73 × 10^-3^–19.17 × 10^-3^) and 9.00 × 10^-3 ^(7.70 × 10^-3^–10.20 × 10^-3^) for African and American strains (Table 1), respectively, and this increase in the level of DNA polymorphism in African strains was statistically significant (*P *= 0.0005, paired sign test). When partitioned by chromosomal location, X-linked loci also show significantly elevated diversity in African strains (17.30 × 10^-3 ^versus 8.15 × 10^-3^, *P *= 0.008, paired sign test), while the increase in African diversity at autosomal loci was only marginally statistically significant (17.79 × 10^-3 ^versus 9.39 × 10^-3^, *P *= 0.07, paired sign test). Based on these estimates of diversity we estimate that the ratio of polymorphism between African and non-African strains is consistently greater than one. When both X-linked and autosomal loci are considered, θAfricanθAmerican
 MathType@MTEF@5@5@+=feaafiart1ev1aaatCvAUfKttLearuWrP9MDH5MBPbIqV92AaeXatLxBI9gBaebbnrfifHhDYfgasaacH8akY=wiFfYdH8Gipec8Eeeu0xXdbba9frFj0=OqFfea0dXdd9vqai=hGuQ8kuc9pgc9s8qqaq=dirpe0xb9q8qiLsFr0=vr0=vr0dc8meaabaqaciaacaGaaeqabaqabeGadaaakeaadaWcaaqaaGGaciab=H7aXnaaBaaaleaacqWGbbqqcqWGMbGzcqWGYbGCcqWGPbqAcqWGJbWycqWGHbqycqWGUbGBaeqaaaGcbaGae8hUde3aaSbaaSqaaiabdgeabjabd2gaTjabdwgaLjabdkhaYjabdMgaPjabdogaJjabdggaHjabd6gaUbqabaaaaaaa@443B@ = 1.96 (95% confidence interval, 1.65–2.33; see Methods), when only X-linked loci are considered, θAfricanθAmerican
 MathType@MTEF@5@5@+=feaafiart1ev1aaatCvAUfKttLearuWrP9MDH5MBPbIqV92AaeXatLxBI9gBaebbnrfifHhDYfgasaacH8akY=wiFfYdH8Gipec8Eeeu0xXdbba9frFj0=OqFfea0dXdd9vqai=hGuQ8kuc9pgc9s8qqaq=dirpe0xb9q8qiLsFr0=vr0=vr0dc8meaabaqaciaacaGaaeqabaqabeGadaaakeaadaWcaaqaaGGaciab=H7aXnaaBaaaleaacqWGbbqqcqWGMbGzcqWGYbGCcqWGPbqAcqWGJbWycqWGHbqycqWGUbGBaeqaaaGcbaGae8hUde3aaSbaaSqaaiabdgeabjabd2gaTjabdwgaLjabdkhaYjabdMgaPjabdogaJjabdggaHjabd6gaUbqabaaaaaaa@443B@ = 2.04 (1.58–2.63), and when only autosomal loci are considered, θAfricanθAmerican
 MathType@MTEF@5@5@+=feaafiart1ev1aaatCvAUfKttLearuWrP9MDH5MBPbIqV92AaeXatLxBI9gBaebbnrfifHhDYfgasaacH8akY=wiFfYdH8Gipec8Eeeu0xXdbba9frFj0=OqFfea0dXdd9vqai=hGuQ8kuc9pgc9s8qqaq=dirpe0xb9q8qiLsFr0=vr0=vr0dc8meaabaqaciaacaGaaeqabaqabeGadaaakeaadaWcaaqaaGGaciab=H7aXnaaBaaaleaacqWGbbqqcqWGMbGzcqWGYbGCcqWGPbqAcqWGJbWycqWGHbqycqWGUbGBaeqaaaGcbaGae8hUde3aaSbaaSqaaiabdgeabjabd2gaTjabdwgaLjabdkhaYjabdMgaPjabdogaJjabdggaHjabd6gaUbqabaaaaaaa@443B@ = 1.90 (1.46–2.39) (Table 2).

**Table 2:** Estimates of Ratios of Diversity

Comparison	Data Included	Ratio^a ^of θ_*s*_	Ratio^a ^of θ_*s *_/Divergence^b^
X/Autosome	African and American populations	0.99 (0.82–1.17)	1.02 (0.79–1.32)
X/Autosome	African strains	0.99 (0.81–1.20)	1.02 (0.77–1.32)
X/Autosome	American strains	0.92 (0.68–1.21)	0.95 (0.64–1.31)
African/American	X-linked and autosomal loci	1.95 (1.65–2.32)	1.95 (1.60–2.36)
African/American	Autosomal loci	1.90 (1.46–2.39)	1.90 (1.42–2.51)
African/American	X-linked loci	2.04 (1.58–2.63)	2.06 (1.46–2.79)

^a ^With (95% confidence interval)

^b ^Divergence with *D. simulans*

Estimates of nucleotide diversity at orthologous pairs of noncoding loci between African and American strains were significantly correlated (Kendall's τ = 0.40; *P *= 0.03). This correlation is at least partly due to mutation rate heterogeneity across loci; if local mutation rate is taken into account by dividing diversity by divergence with *D. simulans*, levels of sequence variation are only marginally correlated between African and American strains (Kendall's τ = 0.31; *P *= 0.07).

Thus, while choosing candidate loci with similar divergence with *D. simulans *accounts for mutation rate heterogeneity to some degree, there remains some residual effect of local mutation rate on locus-specific patterns of polymorphism. To take into account the role of this mutation rate variation in nucleotide polymorphism levels, we divided our diversity estimates by divergence with *D. simulans*. These normalized estimates of diversity are also significantly higher in African versus North American strains overall (3.34 × 10^-1 ^versus 1.72 × 10^-1^, *P *= 0.0005, paired sign test) as well as for X-linked loci (3.38 × 10^-1 ^versus 1.67 × 10^-1^, *P *= 0.02, paired sign test), and marginally significantly higher for autosomal loci (3.35 × 10^-1 ^versus 1.79 × 10^-1^, *P *= 0.07, paired sign test) (Table 1). Using these scaled levels of diversity to estimate the ratio of sequence polymorphism between ancestral and derived populations yields values significantly exceeding one in all cases; the ratios of African to American corrected diversities are 1.95 (1.60–2.36), 1.90 (1.42–2.51), and 2.06 (1.46–2.78) for all loci, autosomal loci, and X-linked loci, respectively (Table 2). The slight differences between the results of these two statistical comparisons of diversity in ancestral and derived populations may reflect the conservative nature of the paired-sign test.

**Figure 2:**
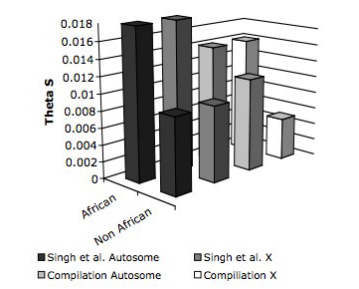
Mean diversities for X-linked and autosomal loci in African and non-African populations of *D. melanogaster*, comparing the Singh *et al*. dataset with the compiled dataset.

Nucleotide polymorphism can also be compared between X-linked and autosomal loci. Average levels of nucleotide polymorphism uncorrected for sequence divergence were 13.28 × 10^-3 ^(95% confidence interval 11.73 × 10^-3^–14.82 × 10^-3^) and 13.10 × 10^-3 ^(11.59 × 10^-3^–14.62 × 10^-3^) (Table 1) for autosomal and X-linked loci when all strains were grouped together. When African and non-African populations were considered separately, X-linked loci and autosomal loci also show similar levels of diversity per site (Figure 2, Table 1). There was no significant difference in estimated polymorphism between X-linked and autosomal loci, either when both populations were considered together or separately (*P *= 0.94, *P *= 0.83, and *P *= 0.46 for both populations, African strains only, and American strains only, respectively, Mann-Whitney U-test). Using these estimates of nucleotide diversity to infer the ratio of X-linked to autosomal variation yielded mean ratios greater than the expected 3/4 under a model of equal numbers of breeding males and females. These ratios were estimated as 0.99 (0.83–1.17), 0.99 (0.81–1.20), and 0.92 (0.68–1.21) for both populations together, African strains, and North American strains, respectively (Table 2). These confidence intervals indicate that we can reject the expected 3/4 ratio of X to autosomal levels of variation for the African population data and for the combined populations.

To again take into account the effects of heterogeneity in local mutation rates, we normalized our estimates of nucleotide diversity by sequence divergence with *D. simulans*. Average levels of normalized nucleotide diversity are 2.53 × 10^-1 ^(2.11 × 10^-1^–3.01 × 10^-1^) and 2.55 × 10^-1 ^(2.12 × 10^-1^–3.08 × 10^-1^) for autosomal and X-linked loci, respectively, when both African and American populations are considered, and this similarity between X-linked and autosomal loci was echoed when African and North American populations were treated separately (Table 1). These differences between the X and the autosomes were not significant in any comparison (*P *= 0.85, *P *= 0.92, and *P *= 0.53 for both populations, African strains, and American strains, respectively, Mann-Whitney U-test). The ratios of normalized diversity levels between X-linked and autosomal loci are 1.03 (0.79–1.32), 1.02 (0.77–1.32), and 0.95 (0.64–1.31) for both populations, African strains and American strains, respectively (Table 2). Much like the uncorrected estimates of nucleotide diversity, all samples show a higher ratio of X-linked to autosomal variation than expected, and for African populations and for the combined dataset we can reject (with 95% confidence) a ratio of 3/4.

### Meta-analysis

To place our results within the context of previous results, we compiled polymorphism data from several published studies (see Methods) (Table 3). When all the data were included, levels of DNA polymorphism were significantly higher in African populations than in non-African populations (Figure 1). Mean diversity in African strains was 13.60 × 10^-3 ^(12.43 × 10^-3^–14.96 × 10^-3^) while mean diversity in non-African strains was 5.96 × 10^-3 ^(4.33 × 10^-3^–6.18 × 10^-3^) (Table 4). Autosomal loci show the same pattern of increased variation in African strains; average polymorphism per site was 13.92 × 10^-3 ^(9.43 × 10^-3^–18.04 × 10^-3^) versus 13.25 × 10^-3 ^(3.90 × 10^-3^–13.52 × 10^-3^) for African and non-African strains, respectively. Sequence variation is significantly higher in African populations for X-linked loci as well, with mean diversities of 13.55 × 10^-3 ^(12.38 × 10^-3^–14.96 × 10^-3^) and 4.73 × 10^-3 ^(3.97 × 10^-3^–5.36 × 10^-3^) for African and non-African strains, respectively (Table 4).

**Table 3:** Data used in the meta analysis published data

Citation	Populations Studied (Number Alleles Sampled)	Sequence Type	Number X-linked Loci	Number Autosomal Loci
Andolfatto 2001	African (> = 2) Non-African (> = 2)	Coding	5	15
Glinka et al. 2003	Zimbabwe (12) Netherlands (12)	Intron Intergenic	105	
Haddrill et al. 2005	Gabon (24) Kenya (24) Zimbabwe (24) Pennsylvania (24) Netherlands (24)	Intron UTR	10	
Kern and Begun 2005	Malawi (5–9)	Intergenic	5	2
Sheldahl et al. 2003	Zimbabwe (4) Crete (10) Massachusetts (10)	Coding	4	4

**Table 4:** Bootstrapped mean diversities and bootstrapped mean diversities normalized by divergence for the meta-analysis dataset

Data Subsample or Ratio	θ_*s*_^a^	θ_*s *_/Divergence^b^
All African	13.60 (12.43–14.96)	2.86 (2.29–2.99)
All Non-African	5.96 (4.33–6.18)	1.32 (0.90–1.44)
All Autosomal	13.59 (8.17–14.61)	3.47 (2.03–4.05)
All X-linked	9.27 (8.56–10.06)	1.93 (1.54–1.96)
African Autosomal	13.92 (9.43–18.04)	3.59 (2.03–5.02)
African X-linked	13.55 (12.38–14.96)	2.74 (2.15–2.80)
Non-African Autosomal	13.25 (3.90–13.52)	3.35 (1.09–3.65)
Non-African X-linked	4.73 (3.97–5.36)	1.04 (0.68–1.17)
All X-linked/Autosomal	0.69(0.59–0.94)	0.57 (0.34–0.71)
African X-linked/Autosomal	1.00 (0.56–1.23)	0.80 (0.21–0.92)
Non-African X-linked/Autosomal	0.37 (0.29–0.60)	0.32 (0.17–0.48)
African/Non-African	2.30 (2.11–2.95)	2.18 (1.64–2.67)
African/Non-African Autosomal	1.09 (0.77–1.87)	1.12 (0.60–1.90)
African/Non-African X-linked	2.88 (2.35–3.38)	2.68 (1.81–3.23)

^a^θ_*s *_× 1000 with (95% confidence interval) for each subsample; ratios of diversity are not multiplied by 1000.

^a^θ_*s *_/Divergence × 10 with (95% confidence interval) for each subsample; ratios are not multiplied by 1000. Divergence as calculated by comparison with *D. simulans*

These differences in mean diversities between populations are recapitulated in our estimates of the ratio of nucleotide diversities in African and non-African strains (Table 4). When all loci are considered, the African/non-African ratio of diversity is significantly greater than one (mean = 2.30, 95% confidence interval = 2.11–2.95). This is also the case when only X-linked loci are included (mean = 2.88, 95% confidence interval = 2.35–3.38). When only autosomal loci are considered the ratio is greater than one but not significantly (mean = 1.09, 95% confidence interval = 0.77–1.87). The lack of difference for autosomal loci might be due to the relative paucity of autosomal loci in this dataset, as there are only 21 autosomal loci contained in this dataset (Table 3). Normalizing diversity levels by divergence further supports higher levels of diversity in African strains, the mean ratio of normalized African to non-African diversity is 2.18 (1.64–2.67) when all loci are included, 1.12 (0.60–1.90) for autosomal loci and 2.68 (1.81–3.23) for X-linked loci (Table 4).

However, factors contributing to sequence variation such as mutation rate variation and recombination were not taken into account when these loci were selected for study. To investigate the relationship between population of origin and sequence variation more thoroughly, we calculated partial correlation coefficients between nucleotide diversity and chromosomal location controlling for the combined effects of local recombination rate and sequence divergence with *D. simulans*. Partial correlation analysis among population (coded as a binary metric with African origin = 1 and non-African origin = 0), nucleotide diversity, recombination rate and sequence divergence revealed an overall strong, positive correlation between population and diversity (Kendall's partial τ = 0.53, P << 0.0001). This trend was echoed when this analysis was restricted to only autosomal or X-linked loci, (Kendall's partial τ = 0.31, τ = 0.61, *P *= 0.02, *P <<*0.0001 for autosomal and X-linked loci, respectively).

This compiled dataset can also be used to specifically compare X-linked and autosomal variation. Overall, levels of diversity (estimated through θ_s_) were 13.59 × 10^-3 ^(8.17 × 10^-3^–14.61 × 10^-3^) and 9.27 × 10^-3 ^(8.56 × 10^-3^–10.06 × 10^-3^) for autosomal and X-linked loci when both ancestral and derived populations were considered. Levels of X-linked and autosomal sequence variation were comparable in African strains, though X-linked variation was markedly depressed relative to autosomal diversity in non-African strains (Figure 2, Table 4). These estimates correspond to ratios of effective population sizes of the X to the autosomes of 0.69 (0.59–0.94) for both populations considered together, 1.00 (0.56–1.23) for African strains and 0.37 (0.296–0.60) for non-African strains. Normalizing diversity values by divergence generates a ratio of X-linked to autosomal diversity of 0.57 (0.34–0.71) for both populations, 0.80 (0.21–0.92) for African strains, and 0.32 (0.17–0.48) for the non-African strains (Table 4).

Accounting for sequence divergence and recombination rate revealed an overall weak yet statistically significant association between autosomal linkage and increased polymorphism (Kendall's partial τ = -0.17, *P *= 0.02, where chromosomal location is coded as a binary metric with autosomal linkage = 0 and X-linkage = 1). This may be due entirely to the sequence data from non-African strains, where a significant association is also found (Kendall's partial τ = -0.28, *P *= 0.0002), as no such relationship found within the data from African strains (Kendall's partial τ = -0.02, *P *= 0.79).

### Simulations

In order to better understand the patterns of variation revealed by these two datasets and to determine if our results are consistent with neutral demographic expectations, we explored the effects of three alternative demographic scenarios on patterns of variability at X-linked and autosomal loci. In the first scenario, we assume that the ancestral population of *D. melanogaster *had similar levels of X-linked and autosomal variation. Under this scenario, a population bottleneck during and after which the ratio of X-linked to autosomal variation and selective patterns remain unchanged should lead to reductions in diversity of similar magnitude on the X chromosome and autosomes. This is because in this model the effective population sizes of the X chromosome and autosomes are equal before, during and after the bottleneck, which leads to identical expected coalescence histories.

In the second scenario, we considered the possibility that patterns of selection or mating structure change during and/or after the population bottleneck. To model the effects of such a change, simulations were conducted using the Thornton and Andolfatto [35] estimated *D. melanogaster *bottleneck parameters (see Methods). Consistent with previous results [36], these simulations suggest that levels of sequence variation on the X chromosome and autosomes can be affected differently by the same demographic event. For instance, if the ratio of the effective number of breeding females to males decreases from 7 (leading to the expected θX−chromosomeθAutosomes
 MathType@MTEF@5@5@+=feaafiart1ev1aaatCvAUfKttLearuWrP9MDH5MBPbIqV92AaeXatLxBI9gBaebbnrfifHhDYfgasaacH8akY=wiFfYdH8Gipec8Eeeu0xXdbba9frFj0=OqFfea0dXdd9vqai=hGuQ8kuc9pgc9s8qqaq=dirpe0xb9q8qiLsFr0=vr0=vr0dc8meaabaqaciaacaGaaeqabaqabeGadaaakeaadaWcaaqaaGGaciab=H7aXnaaBaaaleaacqWGybawcqGHsislcqWGJbWycqWGObaAcqWGYbGCcqWGVbWBcqWGTbqBcqWGVbWBcqWGZbWCcqWGVbWBcqWGTbqBcqWGLbqzaeqaaaGcbaGae8hUde3aaSbaaSqaaiabdgeabjabdwha1jabdsha0jabd+gaVjabdohaZjabd+gaVjabd2gaTjabdwgaLjabdohaZbqabaaaaaaa@4CD5@ of 1 in Africa) in the pre-bottleneck population to 1 during and after the bottleneck, the expected θX−chromosomeθAutosomes
 MathType@MTEF@5@5@+=feaafiart1ev1aaatCvAUfKttLearuWrP9MDH5MBPbIqV92AaeXatLxBI9gBaebbnrfifHhDYfgasaacH8akY=wiFfYdH8Gipec8Eeeu0xXdbba9frFj0=OqFfea0dXdd9vqai=hGuQ8kuc9pgc9s8qqaq=dirpe0xb9q8qiLsFr0=vr0=vr0dc8meaabaqaciaacaGaaeqabaqabeGadaaakeaadaWcaaqaaGGaciab=H7aXnaaBaaaleaacqWGybawcqGHsislcqWGJbWycqWGObaAcqWGYbGCcqWGVbWBcqWGTbqBcqWGVbWBcqWGZbWCcqWGVbWBcqWGTbqBcqWGLbqzaeqaaaGcbaGae8hUde3aaSbaaSqaaiabdgeabjabdwha1jabdsha0jabd+gaVjabdohaZjabd+gaVjabd2gaTjabdwgaLjabdohaZbqabaaaaaaa@4CD5@ ratio in the derived population is reduced to 0.75.

Finally, we considered the possibility that the θX−chromosomeθAutosomes
 MathType@MTEF@5@5@+=feaafiart1ev1aaatCvAUfKttLearuWrP9MDH5MBPbIqV92AaeXatLxBI9gBaebbnrfifHhDYfgasaacH8akY=wiFfYdH8Gipec8Eeeu0xXdbba9frFj0=OqFfea0dXdd9vqai=hGuQ8kuc9pgc9s8qqaq=dirpe0xb9q8qiLsFr0=vr0=vr0dc8meaabaqaciaacaGaaeqabaqabeGadaaakeaadaWcaaqaaGGaciab=H7aXnaaBaaaleaacqWGybawcqGHsislcqWGJbWycqWGObaAcqWGYbGCcqWGVbWBcqWGTbqBcqWGVbWBcqWGZbWCcqWGVbWBcqWGTbqBcqWGLbqzaeqaaaGcbaGae8hUde3aaSbaaSqaaiabdgeabjabdwha1jabdsha0jabd+gaVjabdohaZjabd+gaVjabd2gaTjabdwgaLjabdohaZbqabaaaaaaa@4CD5@ ratio in the ancestral population of the European and North American populations is substantially different from one. This requires either that the ancestral population has not in fact been sampled in any of the studies of African populations, or that extant sequence variation in African strains are not reflective of ancestral levels of diversity. If θX−chromosomeθAutosomes
 MathType@MTEF@5@5@+=feaafiart1ev1aaatCvAUfKttLearuWrP9MDH5MBPbIqV92AaeXatLxBI9gBaebbnrfifHhDYfgasaacH8akY=wiFfYdH8Gipec8Eeeu0xXdbba9frFj0=OqFfea0dXdd9vqai=hGuQ8kuc9pgc9s8qqaq=dirpe0xb9q8qiLsFr0=vr0=vr0dc8meaabaqaciaacaGaaeqabaqabeGadaaakeaadaWcaaqaaGGaciab=H7aXnaaBaaaleaacqWGybawcqGHsislcqWGJbWycqWGObaAcqWGYbGCcqWGVbWBcqWGTbqBcqWGVbWBcqWGZbWCcqWGVbWBcqWGTbqBcqWGLbqzaeqaaaGcbaGae8hUde3aaSbaaSqaaiabdgeabjabdwha1jabdsha0jabd+gaVjabdohaZjabd+gaVjabd2gaTjabdwgaLjabdohaZbqabaaaaaaa@4CD5@ was less than 1 in the ancestral population, a bottleneck should reduce X-linked variation to a greater extent. Simulation results indicate that if, for instance, θX−chromosomeθAutosomes
 MathType@MTEF@5@5@+=feaafiart1ev1aaatCvAUfKttLearuWrP9MDH5MBPbIqV92AaeXatLxBI9gBaebbnrfifHhDYfgasaacH8akY=wiFfYdH8Gipec8Eeeu0xXdbba9frFj0=OqFfea0dXdd9vqai=hGuQ8kuc9pgc9s8qqaq=dirpe0xb9q8qiLsFr0=vr0=vr0dc8meaabaqaciaacaGaaeqabaqabeGadaaakeaadaWcaaqaaGGaciab=H7aXnaaBaaaleaacqWGybawcqGHsislcqWGJbWycqWGObaAcqWGYbGCcqWGVbWBcqWGTbqBcqWGVbWBcqWGZbWCcqWGVbWBcqWGTbqBcqWGLbqzaeqaaaGcbaGae8hUde3aaSbaaSqaaiabdgeabjabdwha1jabdsha0jabd+gaVjabdohaZjabd+gaVjabd2gaTjabdwgaLjabdohaZbqabaaaaaaa@4CD5@ was 0.75 in the ancestral population, a bottleneck should reduce the expected θX−chromosomeθAutosomes
 MathType@MTEF@5@5@+=feaafiart1ev1aaatCvAUfKttLearuWrP9MDH5MBPbIqV92AaeXatLxBI9gBaebbnrfifHhDYfgasaacH8akY=wiFfYdH8Gipec8Eeeu0xXdbba9frFj0=OqFfea0dXdd9vqai=hGuQ8kuc9pgc9s8qqaq=dirpe0xb9q8qiLsFr0=vr0=vr0dc8meaabaqaciaacaGaaeqabaqabeGadaaakeaadaWcaaqaaGGaciab=H7aXnaaBaaaleaacqWGybawcqGHsislcqWGJbWycqWGObaAcqWGYbGCcqWGVbWBcqWGTbqBcqWGVbWBcqWGZbWCcqWGVbWBcqWGTbqBcqWGLbqzaeqaaaGcbaGae8hUde3aaSbaaSqaaiabdgeabjabdwha1jabdsha0jabd+gaVjabdohaZjabd+gaVjabd2gaTjabdwgaLjabdohaZbqabaaaaaaa@4CD5@ ratio to 0.58 in the derived populations.

## Discussion

*Drosophila melanogaster *is a human commensal, and has a cosmopolitan distribution [1]. This species is thought to have originated in Africa, and the demographic history of *D. melanogaster *is marked by an expansion out of Africa into Europe and Asia, followed by the colonization of the Americas [1]. Colonization events are generally comprised of a population bottleneck during migration followed by population expansion in a new habitat, and these phases of the colonization process may have profound consequences for within-population variability in the derived population relative to the ancestral population. Population bottlenecks are expected to depress levels of standing genetic variation [22,36]. The numbers of breeding males and females involved in the founding event can also cause the levels of diversity to differ between the X chromosome and the autosomes [36]. In addition, adaptation to a novel habitat can reduce levels of diversity at selected sites as well as at nearby linked sites [37]; this effect may manifest differentially on the X versus the autosomes if rates of adaptation differ systematically between these chromosome sets.

Because both demographic and selective models predict a reduction in population variability in derived populations, it is very challenging to disentangle the roles of these two forces (for review see [38]). Distinguishing between purely selective and purely demographic models has often relied on the inference that because population bottlenecks affect the entire genome, this demographic event should systematically reduce variation at a genomic scale. In contrast, because adaptive evolution is a locus-specific force, repeated bouts of positive selection due to local adaptation should result in extreme reductions in polymorphism only at selected loci. However, the inherent stochasticity in population bottlenecks can lead to substantial variation across loci in the reduction of nucleotide diversity due to the bottleneck, further complicating the distinction between the effects of demography and natural selection [39]. With a 1:1 sex ratio, the effective size of the X chromosome is 3/4 that of the autosomes, which results in differences in scaling time between the X and the autosomes. When this difference in scaling is properly accounted for, the population bottleneck, although less recent on the X chromosome, becomes more severe (in that it has a smaller minimal size and is effectively longer) on the X chromosome than it is on the autosomes, which leads to a greater reduction of diversity at X-linked loci relative to that found at autosomal loci.

### Comparing patterns of variability between the two datasets

We can directly compare estimates of diversity between the intergenic loci presented here and the data contained in the meta-analysis to identify those populations and/or regions which appear to differ the most dramatically between the two datasets. Given mutation rate variation, the most appropriate metric is polymorphism normalized by divergence. There are no significant differences between the ratio of ancestral to derived (normalized) diversity between the two datasets for either X-linked or autosomal loci (Table 2, Table 4), and both datasets do support a reduction in variation in the non-African strains, which is consistent with a population bottleneck. In African populations, overlapping confidence intervals of the normalized estimates of diversity between the two datasets for both autosomal and X-linked loci (Table 1, Table 4) suggest that these estimates of diversity are similar between the two sets of data. Though the mean estimates of normalized diversity for autosomal regions in non-African populations do differ considerably between the two datasets (3.35 versus 1.79 for the compiled dataset and intergenic dataset, respectively), these estimates also show overlapping confidence intervals between the two datasets, suggesting that the intergenic loci presented here have levels of diversity comparable to those primarily coding regions in the compiled dataset. However, this lack of statistical significance could also be due to lack of power, given that there are only 8 loci in the intergenic dataset and 21 loci in the compiled dataset. In contrast, the two datasets do differ significantly with respect to levels of variation at X-linked loci in non-African populations, with greatly reduced normalized estimates of diversity in the compiled dataset relative to the intergenic dataset. Thus, while the majority of the estimates of diversity are consistent across both datasets, X-linked diversity in derived populations show marked differences between the intergenic loci and the compiled loci.

### X-linked and autosomal variation in African populations

Both the newly collected data and the meta-analysis indicate that θX−chromosomeθAutosomes
 MathType@MTEF@5@5@+=feaafiart1ev1aaatCvAUfKttLearuWrP9MDH5MBPbIqV92AaeXatLxBI9gBaebbnrfifHhDYfgasaacH8akY=wiFfYdH8Gipec8Eeeu0xXdbba9frFj0=OqFfea0dXdd9vqai=hGuQ8kuc9pgc9s8qqaq=dirpe0xb9q8qiLsFr0=vr0=vr0dc8meaabaqaciaacaGaaeqabaqabeGadaaakeaadaWcaaqaaGGaciab=H7aXnaaBaaaleaacqWGybawcqGHsislcqWGJbWycqWGObaAcqWGYbGCcqWGVbWBcqWGTbqBcqWGVbWBcqWGZbWCcqWGVbWBcqWGTbqBcqWGLbqzaeqaaaGcbaGae8hUde3aaSbaaSqaaiabdgeabjabdwha1jabdsha0jabd+gaVjabdohaZjabd+gaVjabd2gaTjabdwgaLjabdohaZbqabaaaaaaa@4CD5@ in African populations is elevated above the expected 0.75 (under an assumption of equal numbers of breeding males and females); the data presented here suggest that the ratio is 0.99 and the estimate of the ratio is nearly identical in the meta-analysis dataset, at 1.00 (Table 2, Table 4). Given that we now have information from a variety of loci including sequences that are both coding and noncoding, located far and close to genes, and represent different types of molecular markers, we believe these results to be robust. Moreover, these surveys utilized strains collected in different parts of Africa (Table 3), which hints at the possibility that these patterns are not restricted to any one particular African population, although given population structure in Africa [40,41] these patterns may be restricted to certain geographic locales within the continent. Finally, our analyses suggest that these results are also robust to variation in recombination rate, levels of selective constraint, and mutation rates.

There are several possible explanations for the increase in X-linked sequence variation relative to expectation. One possibility is that the variance of reproductive success in *D. melanogaster *males is larger than that in females. Recall that the effective population size of an autosomal locus (NeA=4NMNFNM+NF
 MathType@MTEF@5@5@+=feaafiart1ev1aaatCvAUfKttLearuWrP9MDH5MBPbIqV92AaeXatLxBI9gBaebbnrfifHhDYfgasaacH8akY=wiFfYdH8Gipec8Eeeu0xXdbba9frFj0=OqFfea0dXdd9vqai=hGuQ8kuc9pgc9s8qqaq=dirpe0xb9q8qiLsFr0=vr0=vr0dc8meaabaqaciaacaGaaeqabaqabeGadaaakeaacqWGobGtdaWgaaWcbaGaemyzau2aaSbaaWqaaiabdgeabbqabaaaleqaaOGaeyypa0ZaaSaaaeaacqaI0aancqWGobGtdaWgaaWcbaGaemyta0eabeaakiabd6eaonaaBaaaleaacqWGgbGraeqaaaGcbaGaemOta40aaSbaaSqaaiabd2eanbqabaGccqGHRaWkcqWGobGtdaWgaaWcbaGaemOrayeabeaaaaaaaa@3D5D@) and the effective size of an X-linked locus (NeX=9NMNF4NM+2NF
 MathType@MTEF@5@5@+=feaafiart1ev1aaatCvAUfKttLearuWrP9MDH5MBPbIqV92AaeXatLxBI9gBaebbnrfifHhDYfgasaacH8akY=wiFfYdH8Gipec8Eeeu0xXdbba9frFj0=OqFfea0dXdd9vqai=hGuQ8kuc9pgc9s8qqaq=dirpe0xb9q8qiLsFr0=vr0=vr0dc8meaabaqaciaacaGaaeqabaqabeGadaaakeaacqWGobGtdaWgaaWcbaGaemyzau2aaSbaaWqaaiabdIfaybqabaaaleqaaOGaeyypa0ZaaSaaaeaacqaI5aqocqWGobGtdaWgaaWcbaGaemyta0eabeaakiabd6eaonaaBaaaleaacqWGgbGraeqaaaGcbaGaeGinaqJaemOta40aaSbaaSqaaiabd2eanbqabaGccqGHRaWkcqaIYaGmcqWGobGtdaWgaaWcbaGaemOrayeabeaaaaaaaa@3F7D@) depends on the effective numbers of breeding males (*N*_*M*_) and breeding females (*N*_*F*_) in the system [42]. When there are equal numbers of breeding males and females, the expected ratio of effective sizes of the X and the autosomes is 3/4. To achieve a X/A ratio of 1, we can calculate from these equations that NMNF
 MathType@MTEF@5@5@+=feaafiart1ev1aaatCvAUfKttLearuWrP9MDH5MBPbIqV92AaeXatLxBI9gBaebbnrfifHhDYfgasaacH8akY=wiFfYdH8Gipec8Eeeu0xXdbba9frFj0=OqFfea0dXdd9vqai=hGuQ8kuc9pgc9s8qqaq=dirpe0xb9q8qiLsFr0=vr0=vr0dc8meaabaqaciaacaGaaeqabaqabeGadaaakeaadaWccaqaaiabd6eaonaaBaaaleaacqWGnbqtaeqaaaGcbaGaemOta40aaSbaaSqaaiabdAeagbqabaaaaaaa@31A2@.

Consequently, as the ratio of effectively reproducing females and males (F/M) increases from 1 to 7, the expected θX−chromosomeθAutosomes
 MathType@MTEF@5@5@+=feaafiart1ev1aaatCvAUfKttLearuWrP9MDH5MBPbIqV92AaeXatLxBI9gBaebbnrfifHhDYfgasaacH8akY=wiFfYdH8Gipec8Eeeu0xXdbba9frFj0=OqFfea0dXdd9vqai=hGuQ8kuc9pgc9s8qqaq=dirpe0xb9q8qiLsFr0=vr0=vr0dc8meaabaqaciaacaGaaeqabaqabeGadaaakeaadaWcaaqaaGGaciab=H7aXnaaBaaaleaacqWGybawcqGHsislcqWGJbWycqWGObaAcqWGYbGCcqWGVbWBcqWGTbqBcqWGVbWBcqWGZbWCcqWGVbWBcqWGTbqBcqWGLbqzaeqaaaGcbaGae8hUde3aaSbaaSqaaiabdgeabjabdwha1jabdsha0jabd+gaVjabdohaZjabd+gaVjabd2gaTjabdwgaLjabdohaZbqabaaaaaaa@4CD5@ ratio increases 0.75 to 1, and can increase to as much as 1.125 as the F/M ratio is elevated even further.

It is also possible that the relative elevation of variability on the X-chromosome is due to different patterns of natural selection acting on the X-linked and autosomal genes. Background selection could in principle generate this pattern as well, as this model predicts higher levels of neutral variation on the X chromosome [43-46]. This is a consequence of the greater efficacy of background selection on X chromosome due to the hemizygosity of the X chromosome in males, which results in a larger proportion of X chromosomes that are free of deleterious mutations. Whether background selection, especially in regions of high recombination, could be sufficiently strong to elevate the θX−chromosomeθAutosomes
 MathType@MTEF@5@5@+=feaafiart1ev1aaatCvAUfKttLearuWrP9MDH5MBPbIqV92AaeXatLxBI9gBaebbnrfifHhDYfgasaacH8akY=wiFfYdH8Gipec8Eeeu0xXdbba9frFj0=OqFfea0dXdd9vqai=hGuQ8kuc9pgc9s8qqaq=dirpe0xb9q8qiLsFr0=vr0=vr0dc8meaabaqaciaacaGaaeqabaqabeGadaaakeaadaWcaaqaaGGaciab=H7aXnaaBaaaleaacqWGybawcqGHsislcqWGJbWycqWGObaAcqWGYbGCcqWGVbWBcqWGTbqBcqWGVbWBcqWGZbWCcqWGVbWBcqWGTbqBcqWGLbqzaeqaaaGcbaGae8hUde3aaSbaaSqaaiabdgeabjabdwha1jabdsha0jabd+gaVjabdohaZjabd+gaVjabd2gaTjabdwgaLjabdohaZbqabaaaaaaa@4CD5@ substantially and, more specifically, from 0.75 to 1 is unclear.

Finally, it is possible that the ratio of X-linked to autosomal diversity is greater than expected because of a reduction in levels of polymorphism on the autosomes rather than an increase in polymorphism on the X chromosome. It is possible that adaptation is more frequent and/or involves adaptive events of greater selective strength on the autosomes than on the X chromosome. This would reduce autosomal variation relative to that on the X chromosome, and is theoretically possible if adaptive evolution operates primarily on standing variation as opposed to novel beneficial mutations [47].

It has also been suggested that a reduction in autosomal variation could result from the presence of polymorphic inversions [22]; because inversion heterozygosity could in theory suppress recombination and thus increase linkage among sites, the reduction in diversity due to selection may be more pronounced in genomic regions with inversions. Moreover, levels of variability within inversions of young to intermediate age are lower than expected [48,49]. Inversions do appear to be more common on the autosomes than on the X chromosome in African populations of *D. melanogaster *[50] which may contribute to a reduction in autosomal diversity relative to expectation in these populations. Consistent with this model, four of the eight autosomal intergenic regions presented here are contained within the breakpoints of five of the large inversions that are frequent in Africa (In(2L)t, In(2R)NS, In(3R)K, In(3R)P and In(3L)P) [51]; levels of polymorphism at these regions is approximately 1/2 that found in the four intergenic regions outside of these known inversions in the African population (data not shown). However, it is important to note that if an inversion is sufficiently old and is maintained as a balanced polymorphism, it may also serve to increase standing diversity, acting as a balancer and thereby preventing the coalescence of inverted and non-inverted chromosomes. Thus, the effects of inversions on levels of molecular variation in Drosophila merits further investigation.

### X-linked and autosomal variation in non-African populations

The results from the non-African populations collected for the present study and from the meta-analysis are more difficult to interpret. Both sets of results support a reduction in diversity in these populations, although the magnitude of the reduction on the X and the autosomes differs substantially between the two datasets. The regions within or nearby genes collated and reanalyzed here argue for a much more severe reduction on the X chromosome than on autosomes (Table 4), which, in addition to being largely consistent with the individual studies in the meta-analysis [4,5,22,23,52], are also consistent with studies of microsatellite variability [29]. In contrast, the analysis of sixteen intergenic regions presented in this paper shows very similar reductions in diversity on the X chromosome and the autosomes. This is primarily due to a greater reduction in X-linked diversity in the compiled dataset, as reductions in diversity at autosomal loci are similar between the two datasets.

To reconcile the differences between these two datasets, we considered several alternative demographic scenarios, and assessed which aspects of the polymorphism data were consistent with each model. First, we considered a model with equal effective population sizes of the X and the autosomes in the ancestral population in combination with a population bottleneck during and after which the ratio of effective sizes of the X to the autosomes is unchanged. Under such a scenario, we expect similar levels of X-linked and autosomal variation in ancestral populations as well as in derived populations. Thus, this model is entirely consistent with the patterns of X-linked and autosomal variability from both African and non-African strains at the intergenic loci presented in the current study, and is similarly consistent with patterns of variation at X-linked and autosomal loci in African populations of the compiled dataset. In this case, the reduced diversity at X-linked loci seen in derived populations in the compiled dataset must be explained. One possibility for the reduction in diversity on the X chromosome in derived strains is genetic hitchhiking; under this model nucleotide diversity on the X is lower than autosomal diversity due to more frequent or more recent selective sweeps on the X chromosome relative to the autosomes, perhaps due to adaptation to temperate climates in these derived strains.

A second plausible demographic model is one in which the ancestral population has similar levels of autosomal and X-linked variation, but that this ratio is shifted towards a higher effective size of the autosomes relative to the X during and after the bottleneck. Under this model, we expect that levels of X-linked diversity will be more severely reduced than levels of diversity on the autosomes; this demographic model is thus consistent with all of the African polymorphism data presented here as well as the non-African polymorphism data presented in the combined dataset.

Therefore, the increased X-linked diversity (or decreased autosomal diversity) in North American populations at the intergenic regions presented here requires explanation. One possibility is that patterns of background selection at intergenic loci have changed between putatively ancestral and derived populations. As described earlier, the greater efficacy of background selection at X-linked loci can increase standing variation in these regions relative to the autosomes, which suggests that if background selection plays a larger role at intergenic loci in derived populations, then the reduction of nucleotide diversity due to background selection would be more pronounced on the autosomes than on the X chromosome.

Finally, we considered a demographic model in which the θX−chromosomeθAutosomes
 MathType@MTEF@5@5@+=feaafiart1ev1aaatCvAUfKttLearuWrP9MDH5MBPbIqV92AaeXatLxBI9gBaebbnrfifHhDYfgasaacH8akY=wiFfYdH8Gipec8Eeeu0xXdbba9frFj0=OqFfea0dXdd9vqai=hGuQ8kuc9pgc9s8qqaq=dirpe0xb9q8qiLsFr0=vr0=vr0dc8meaabaqaciaacaGaaeqabaqabeGadaaakeaadaWcaaqaaGGaciab=H7aXnaaBaaaleaacqWGybawcqGHsislcqWGJbWycqWGObaAcqWGYbGCcqWGVbWBcqWGTbqBcqWGVbWBcqWGZbWCcqWGVbWBcqWGTbqBcqWGLbqzaeqaaaGcbaGae8hUde3aaSbaaSqaaiabdgeabjabdwha1jabdsha0jabd+gaVjabdohaZjabd+gaVjabd2gaTjabdwgaLjabdohaZbqabaaaaaaa@4CD5@ ratio in the ancestral population is less than 1, coupled with a population bottleneck. This scenario also leads to more extreme reductions of diversity at X-linked loci, which is what is observed in the non-African strains at the loci in the combined dataset. It is not clear whether biologically plausible scenarios under this model can generate a θX−chromosomeθAutosomes
 MathType@MTEF@5@5@+=feaafiart1ev1aaatCvAUfKttLearuWrP9MDH5MBPbIqV92AaeXatLxBI9gBaebbnrfifHhDYfgasaacH8akY=wiFfYdH8Gipec8Eeeu0xXdbba9frFj0=OqFfea0dXdd9vqai=hGuQ8kuc9pgc9s8qqaq=dirpe0xb9q8qiLsFr0=vr0=vr0dc8meaabaqaciaacaGaaeqabaqabeGadaaakeaadaWcaaqaaGGaciab=H7aXnaaBaaaleaacqWGybawcqGHsislcqWGJbWycqWGObaAcqWGYbGCcqWGVbWBcqWGTbqBcqWGVbWBcqWGZbWCcqWGVbWBcqWGTbqBcqWGLbqzaeqaaaGcbaGae8hUde3aaSbaaSqaaiabdgeabjabdwha1jabdsha0jabd+gaVjabdohaZjabd+gaVjabd2gaTjabdwgaLjabdohaZbqabaaaaaaa@4CD5@ ratio as low as 0.37, as observed in the meta-analysis dataset, but these simulation results suggest that an exaggerated depression of diversity at X-linked loci can be at least partly explained by demographic scenarios. This model thus requires that either extant levels of sequence variation in African strains are not reflective of ancestral polymorphism levels or that the ancestral populations of this species have not been sampled in the datasets presented here. Moreover, this model cannot explain the similar levels of sequence variation at X-linked and autosomal loci found at the intergenic regions collected for the current study; the excess of X-linked polymorphism or dearth of autosomal variation in these regions could result from a change in the patterns of background selection during and after the population bottleneck.

It is indeed difficult to distinguish between these alternative explanations for the differences between the datasets presented here. Based on the currently available data, however, we believe the demographic model of a female-biased ancestral sex ratio coupled with a population bottleneck that retains this ratio to be most appropriate for two reasons. First, the increased levels of diversity at the intergenic loci chosen in the current study as compared to the levels of diversity at loci in the compiled dataset (Figure 2) are consistent with reduced effects of background selection and genetic hitchhiking in these regions. This suggests that patterns of variation at these regions are more likely to reflect demographic rather than selective processes. Second, to the extent that extant levels of sequence variation in African strains are indicative of ancestral levels of polymorphism, the comparable levels of X-linked and autosomal nucleotide diversity in sampled African populations of *D. melanogaster *are suggestive of an unequal sex ratio in ancestral populations of this species.

## Conclusion

On balance, therefore, we believe that given the currently available data, a demographic model with unequal numbers of breeding males and females may be the most appropriate explanation for the current data. This model is suggestive of a ratio of breeding females to breeding males of 7:1, and while it is not beyond reason that males could have a larger variance in reproductive success than females in Drosophila, it is not clear whether a ratio of 7:1 is realistic. Indeed, it is challenging to assess the plausibility of this ratio, given the lack of relevant parameter estimates from natural populations of this species.

It is important to note that since this analysis was undertaken, additional polymorphism datasets have been made available [3,53]. We believe our results are likely robust to the inclusion of the new X-linked loci presented in [3], as our current meta-analysis includes almost one-half of the loci in the more recent study, and no major differences between the initial [4] and later [3] X-linked datasets were reported. In addition, the recent survey of autosomal polymorphism, while supportive of a female-biased sex ratio in Africa, seem to suggest a male-biased sex ratio in Europe [53]. Thus, it remains to be seen whether the patterns we observe are characteristic of the genome and moreover, whether the patterns in non-African strains vary with population of origin; as more polymorphism data becomes available, we will have increasing power to test this explicitly.

The demographic model we present assumes that the effective numbers of breeding males and females remains unchanged throughout the demographic history of this species, and can fully account for the patterns of diversity at X-linked and autosomal loci in African and North American strains of *D. melanogaster *from the intergenic regions presented here. The exaggerated reduction of diversity of X-linked regions in derived populations contained within the compiled dataset unfortunately remains difficult to explain. One possibility is that this reduction is due to increased hitchhiking on the X chromosome in these populations; that divergence at X-linked loci is significantly higher than autosomal divergence in the compiled dataset (*P *= 0.002, Mann-Whitney U-test) supports this hypothesis.

Importantly, the hitchhiking aspect of this model must be discussed in the context of several previous observations. First, X-linkage does not systematically increase rates of molecular evolution; while some previous studies do support a faster-X model [54,55], the most recent and most comprehensive test of this model shows no evidence for faster-X evolution [56]. The polymorphism data presented here, however, hint at the possibility that the effects of genetic hitchhiking may be more pronounced on the *D. melanogaster *X chromosome than on the autosomes in association with population expansion to new habitats. These results could be reconciled either by suggesting that the increase in rates of positive selection is too episodic to elevate the rate of evolution overall on the X chromosome, or that X-linked loci are also subject to stronger purifying selection. More frequent fixation of allelic variants by positive selection could be balanced by a lower rate of fixation of slightly deleterious alleles on the X chromosome.

Second, if our results are indeed reflective of recent adaptive events on the X chromosome, we do expect to observe the signature of selective sweeps on this chromosome. While previous studies have identified regions of the X-chromosome that have patterns of polymorphism consistent with selective sweeps, patterns of variation at these regions are also largely consistent with demographic effects (for review see [57]). Indeed, identifying loci whose patterns of sequence variation cannot be explained by demography alone depends on the demographic model employed and the population bottleneck parameters (for review see[57]). Thus, further investigation of the effects of population bottlenecks under a variety of bottleneck parameters on X-linked and autosomal variation, as well as additional examination of what bottleneck parameters may be appropriate for *D. melanogaster *may be warranted.

Another observation that merits discussion is the estimation of selective constraint in the *D. melanogaster *genome. Recent studies of constraint in Drosophila appear to provide evidence that in addition to genic regions such as introns and synonymous sites, intergenic regions also appear to be subject to selective constraint [58-60]. This bears particular relevance given that our simulations are based on the evolution of neutral sequences; to the extent that none of the regions presented here, intergenic or otherwise, are truly neutral, the results from our simulations may not prove accurate. Consequently, modeling the joint effects of demography and selection on X-linked and autosomal variation should prove illuminating.

Importantly, this model of higher rates of adaptation at X-linked regions in close proximity to genes in derived populations makes several predictions that can be explicitly tested in the future. For instance, a model of recurrent selection will only produce decreased nucleotide variation on the X chromosome in a species such as *D. melanogaster *(with recombination in females only) if natural selection acts on new mutations and these new mutations are at least partially recessive [61]. This is of particular importance given that the majority of polymorphic sites in non-African strains are polymorphic in African strains as well [4]. However, there are certainly mutations that are novel to derived strains of *D. melanogaster*, and given a specific scenario of the population expansion out of Africa, it may be possible to theoretically estimate the beneficial mutation rate required to produce such a depression of X-linked polymorphism and assess whether such estimates are biologically plausible.

In addition, this model predicts systematic differences in the site-frequency spectrum between the X and the autosomes; more frequent or more recent episodes of adaptive evolution should produce a skew in the frequency spectrum towards an excess of rare variants on the X relative to the autosomes [36]. Comprehensive analysis of the frequency spectrum of segregating variants at a large collection of X-linked and autosomal loci will thus shed light on the potential relevance of this explanation.

Further, this model predicts that rates of adaptation are higher in derived populations than in ancestral populations. Given the currently available data, this does not appear to be the case, at least for X-linked loci[57], but additional data would certainly make the estimates of the rate of adaptive substitution more accurate, and may reveal differences between ancestral and derived populations, or between X-linked and autosomal loci. In addition, this model predicts that there should be marked divergence between African and non-African strains, particularly for X-linked loci, due to increased rates of adaptation in derived strains. Further investigation of this question will also shed light on the viability of this model.

Moreover, given the effects of genetic hitchhiking on sequence diversity, this model predicts that bottleneck parameters estimated using X chromosome data will differ from those parameters estimated using autosomal data. The most recent estimates of these parameters from *D. melanogaster *[35,62] are indeed based on a dataset which, while wonderfully rich, is comprised entirely of X-linked loci. There is little question that this dataset of X-linked loci is the most comprehensive dataset available for such analyses, but if there is a greater incidence of hitchhiking on the X in derived populations, the severity of the bottleneck may have been overestimated. A perhaps fruitful avenue of future inquiry would be to reevaluate the estimation of bottleneck parameters using only autosomal data, should such data become available.

Finally, if this model is correct, it will have a number of important implications. First, patterns of variation of X-linked and autosomal diversity depend heavily on sequence type, which suggests that the evolutionary forces responsible for the generation and maintenance of sequence diversity are heterogeneous across the genome, with marked differences between sequences that are either genic or in close proximity to genic sequence and regions that are distant to genes. Second, the similarity of the effective population sizes of the X chromosome and autosomes in the African populations should make natural selection more effective for X-linked loci in general, at least in these populations. For example, for identical co-dominant mutations on the X chromosome and the autosomes we expect that the absolute value of the effective strength of selection (*N*_*e*_*s*) to be 33% larger on the X chromosome [30]. It is possible that this effect is sufficient to account for the higher codon bias of the X-linked genes in *D. melanogaster *[30-32]. The higher codon bias of the X-linked genes in *D. pseudoobscura *[30] might imply that effective population size of the X chromosomes in general is higher than 3/4 of the autosomal effective population size throughout the Drosophila genus; this effect could be due to a generally higher variance of reproductive success in males than females throughout Drosophila.

## Methods

### Selection of Loci

We selected eight autosomal and eight X-linked loci from noncoding regions of the *D. melanogaster *genome. We began with all noncoding loci (not contained within the coordinates of coding sequences as reported in version 3.1 of the *D. melanogaster *genome) that were at least 5 kb from annotated genes in both directions, as measured from the midpoint of the noncoding locus to the edge (5' or 3' end, depending on the genic orientation) of the nearest coding sequence. We restricted these candidates to those with no significant BLASTX hits with an E-value cutoff of 0.001 in the hopes of restricting ourselves to loci that were truly noncoding. We further limited ourselves to loci located in regions of high recombination, with estimates of local recombination rate between 3.25 and 3.5 cM/Mb [33] (see also [63]). We allowed for a maximum GC content of 45%, and used only regions for which there was an Exelixis deletion strain encompassing the entire locus available (see below). From this list of candidate loci, we selected two regions from each of the four major autosomal chromosome arms (2L, 2R, 3L, and 3R) as well as eight regions from the X chromosome, maximizing the physical distance among loci for each chromosome arm. Finally, we computed sequence divergence between each locus and the orthologous locus in *D. simulans *(based on the consensus sequence) and used these values to identify regions that were evolving as close as possible to expectation under neutrality. We chose X-linked and autosomal loci such that the distributions of divergence values for our autosomal and X-linked loci were not significantly different (*P *= 0.59, Mann-Whitney U-test) to control for variation in mutation rate.

### Fly Strains and Genomic DNA Extraction

We have used 12 North American strains (6 from North Carolina (gift from G. Gibson) and 6 from Davis, California (gift from S. Nuzhdin)) and 12 African strains from Malawi. Because the American strains had been passed through > 30 generations of brother-sister mating (S. Nuzhdin and G. Gibson, personal communications), there was very little nucleotide variability within in each strain. We therefore assumed and then confirmed that individuals from these strains were nearly homozygous at each of our studied loci.

In contrast, the strains from Malawi had a great deal of nucleotide diversity and measures were taken to effectively homozygose these strains at each of our eight autosomal loci. We took advantage of the *D. melanogaster *deficiency collection created by Exelixis [64] and purchased eight deficiency strains, each of which had a deletion at one of the studied autosomal loci. We then crossed each of our twelve Malawi strains with each of the 8 deletion strains and used the appropriate 96 F1's for our analyses. These F1's had only a single copy of one of the parental Malawi haplotypes at the studied locus.

We extracted genomic DNA from a single male from each of the strains according to a protocol described by G. Gloor and W. Engels (personal communication). Each fly was crushed with the end of a pipette tip and subsequently immersed in a buffered solution (10 mM Tris-Cl pH 8.2, 1 mM EDTA, 25 mM NaCl, 200 μg/mL proteinase K). This solution was incubated at 37°C for 30 minutes and then at 95°C to inactivate the proteinase K.

### Primer Design, PCR Amplification and Sequencing

We designed primers for our sixteen loci using Primer3 [65] and ensured that each primer pair would produce a unique amplicon using Virtual PCR [66]. Primer sequences are available upon request. Our PCR reactions were 20 μL and contained 10 μL of RedTaq Ready Mix (Qiagen), 1 μL of each 20 μM primer, 1 μL of genomic DNA and 7 μL water. We used a touchdown PCR program to amplify our loci to facilitate direct sequencing. The amplifying conditions were as follows: 94°/30 seconds (s), T_m_/30s, 72°/30s, where T_m _began at 62° and was decreased by 0.5° each cycle, followed by 20 cycles of 94°/30s, 55°/30s, 72°/30s, with a final 10 minute extension period at 72°. These PCR products were sequenced directly at Genaissance Pharmaceuticals using our amplifying primers.

### Data Analysis

Sequences were manually aligned using Sequencher version 4.2.2, and values of Tajima's D were calculated using DnaSP version 3.1. Statistical analyses were performed in Statview version 5.0. Mean levels of nucleotide diversity (θ_s_) and confidence intervals for these estimates were obtained by bootstrapping over all sites in each respective dataset in order to compare diversity across loci and populations; bootstrapping was done with scripts that were developed in-house for the purposes of this project. The values reported are the means of their respective bootstrap distributions. Each confidence interval derives from 1000 bootstrap replicates in the case of the intergenic dataset and 10,000 replicates in the case of the compiled dataset.

### Meta-Analysis

We also compiled polymorphism data from several previously published results. Included in our meta-analysis are seven intergenic loci from Kern and Begun [5], 20 coding regions from Andolfatto [22], 10 coding regions from Haddrill et al. [52], 105 noncoding regions from Glinka et al. [4] and 8 coding regions from Sheldahl et al. [23] (Table 3). These data include both X-linked and autosomal loci. For the purpose of calculating θ_*s *_and θ_*s *_/divergence, loci which were available in more than one strain were given equal weights, such that total weight of the locus was 1. Both the observed values and bootstrap values of θ_*s *_and θ_*s *_/divergence were calculated using these weights. Estimates of polymorphism were taken directly from the literature, as were estimates of divergence with *D. simulans *if reported. If divergence estimates were not presented, we retrieved the studied sequences and calculated divergence against the consensus sequence of the *D. simulans *genome. We also calculated local recombination rate at each locus following a procedure detailed previously [33].

### Simulations

We conducted coalescent simulations to estimate the expected effect of a bottleneck under different demographic scenarios. Simulations were carried out using *ms *[67]. The bottleneck parameters for the X chromosome are taken from Thornton and Andolfatto [35]. Specifically we take the population bottleneck on the X chromosome to last from 0.022 to 0.0042 in units of the expected time to coalescence between two alleles. During the bottleneck the effective population size is reduced to 0.029 of that before and after the bottleneck. The simulation command line for the scenario in which F/M ratio goes from 7 in the ancestral population to 1 during and after the bottleneck is:

X: ms 20 100000 -t 40.0 -r 40.0 10000 -eN 0.0042 0.029 -eN 0.022 1.0

A: ms 20 100000 -t 53.4 -r 53.4 10000 -eN 0.00315 0.029 -eN 0.0165 0.75

The simulation code for the scenario in which F/M ratio stays at 1 throughout the simulations is:

X: ms 20 10000 -t 30.0 -r 30.0 10000 -eN 0.0042 0.029 -eN 0.022 1.0

A: ms 20 10000 -t 40.0 -r 40.0 10000 -eN 0.00315 0.029 -eN 0.0165 1.0

## Authors' contributions

NDS and DAP conceived the idea for this project. JMM and DAP carried out coalescent simulations. NDS, JMM and DAP participated in the analysis and contributed to the interpretation of the simulation results. NDS, JMM, JDJ and DAP contributed to the interpretation of the empirical results. NDS, JMM, and DAP were responsible for the writing of this manuscript, and NDS, JMM, JDJ and DAP were responsible for the editing of this manuscript. All authors have given their final approval for its publication.
